# Combinations of propolis and Ca(OH)2 in dental pulp capping treatment for the stimulation of reparative dentin formation in a rat model

**DOI:** 10.12688/f1000research.22409.1

**Published:** 2020-04-29

**Authors:** Retno Pudji Rahayu, Nirawati Pribadi, Ira Widjiastuti, Nur Ariska Nugrahani

**Affiliations:** 1 Department of Oral and Maxillofacial Pathology, Faculty of Dentistry, Airlangga University, Surabaya, East Java, 60131, Indonesia; 2 Department of Conservative Dentistry, Faculty of Dentistry, Airlangga University, Surabaya, East Java, 60131, Indonesia; 3 Immunology Study Program, Airlangga University, Surabaya, East Java, 60131, Indonesia

**Keywords:** Propolis, Ca (OH)2, IL-10, IL-8, TGF-ß, VEGF dan TLR-2

## Abstract

**Background: **Caries in the dental pulp result in inflammation and damage to the pulp tissue. During inflammation of the pulp, various inflammatory mediators and growth factors are released, including IL-8, IL-10, TLR-2, VEGF and TGF-β through the NF-kB pathway. In the present study, therapy for pulpal caries was performed through pulp capping by giving a combination of propolis and calcium hydroxide (Ca(OH)2). This treatment was expected to stimulate the formation of reparative dentin as an anti-inflammatory material to prevent pulp tissue damage.

**Methods:** 28 Wistar rats were divided into four groups and treated with Ca(OH)2 with or without the addition of propolis for either 7 or 14 days. Immunohistochemical examination was used to determine the expression of IL-8, IL-10, TLR-2, VEGF, TGF-β in the four treatment groups.

**Results: **The group treated with a combination of propolis and Ca(OH)2 for 7 days showed that the expression of IL-10, IL-8, TLR-2, VEGF, TGF-β increased significantly compared to the treatment group treated with only Ca(OH)2. The expression of IL-10, TLR-2, TGF-β, VEGF increased in the treatment group treated with propolis and Ca(OH)2 for 14 days, while the expression of IL-8 in the decreased significantly.

**Conclusions: **Administration of a combination of propolis and Ca(OH)2 has efficacy in the pulp capping treatment process because it has anti-bacterial and immunomodulatory properties. The results show that it is able to stimulate the process of pulp tissue repair through increased expression of IL-10, TGF-β, VEGF, TLR -2 and decreased expression of IL-8.

## Introduction

Caries in the dental pulp result in inflammation and damage to the pulp tissue. During inflammation of the pulp, inflammatory mediators, including cytokines, regulate the activity of the immune response to local and systemic inflammation against the external stimuli ^1– 3^. NF-kB activation due to injury causes transcription and translational processes in cells, which results in pro-inflammatory cytokine production, such as IL-1β, IL-6 and TNF-α, initiating the immunological process that aids pulp tissue in overcoming inflammation ^3^. NF-kB is considered as a prototypical inflammatory pathway and is used to target anti-inflammatory drugs, thus the expression of NF-kB can be investigated to measure the inhibition of proinflammatory cytokine production ^3, 4^.

Calcium hydroxide (Ca(OH) _2_) is the gold standard for pulp capping treatment in tooth pulp inflammation but has a high pH (pH 12.5), which causes pulp tissue necrosis in direct contact with Ca(OH) _2_^5^. Ca(OH) _2_ plays a role in the formation of reparative dentine, apexification, and intracanal medicament. It has anti-microbial properties (able to neutralize microbial products), inhibits root resorption and induces the formation of hard tissue ^6^. The application of Ca(OH) _2_ with a certain concentration causes fibroblast cell death resulting in damage to the pulp tissue ^7^. In addition, use of Ca(OH) _2_ in long term can cause physical changes; reparative dentine formation is not dense enough due to dentin bridge discontinuity leading to an area of necrosis called a "tunnel defect", whichcauses dentists to search for alternative materials, including natural herbal-based ingredients ^8^. Propolis is a natural material produced by bees, consisting of balsam resin, beeswax, essential oils, and pollen from other organic materials. It may have a role in the process of pulp capping treatment and the healing process, due to its anti-bacterial, anti-fungal, anti-virus, anti-tumor, anti-oxidation, and immunomodulatory properties ^9^. It has been reported that toxicity of propolis to fibroblast cells is low and is able to inhibit apoptosis and stimulate fibroblast cell proliferation ^10^. In addition, propolis may be used as an alternative herbal ingredient for pulp capping treatment because it can reduce inflammation in pulp exposed to cariogenic material ^11^. The inhibition of NF-kB by the active component of propolis causes a decrease in the expression of proinflammatory cytokine genes, such as IL-1, IL-6, IL-8, and TNF-α, leading to downregulation of TLR-2 in dental pulp ^12, 13^. The combination of Ca(OH) _2_ with propolis does not cause a toxic reaction, which was shown when evaluating the biocompatibility of Ca(OH) _2_ and propolis in the subcutaneous tissue of rats. It was found that both materials were able to reduce inflammation significantly through the role of IL-10, TGF-β, VEGF and was biocompatible with rat connective tissue ^11, 14^. Propolis contains caffeic acid phenethyl ester (CAPE), showing that propolis can function as an anti-inflammatory. However, to date research on pulp capping treatment for the stimulation of reparative dentin formation with a combination of Ca(OH) _2_ and propolis in an immunopathological study has not yet been performed. Therefore, this study aimed to find out if there was increased expression of IL-10, VEGF, TGF-β and TLR-2 as well as a decrease in pro-inflammatory cytokines IL-8 when using Ca(OH) _2_ in combination with propolis.

## Methods

### Ethics statement

All procedures performed in this study were ethical, and the study was approved by the Ethics Commission of the Dental and Oral Hospital of the Faculty of Dentistry, Airlangga University (002/HRECC.DHAU/XI/2019) with principal researcher as RPR. The study took place at the Department of Biochemistry, Medical Faculty, Airlangga University and Department of Biochemistry, Medical Faculty, Brawijaya University, which were certified to be ethical. All efforts were made to ameliorate any suffering of animals with the use of local anesthesia (lidocaine with Citoject syringe) to decrease pain in this treatment.

### Animals

Adult male Wistar rats aged 16–18 weeks, weight 150–250 grams, with healthy teeth and no caries, were kept in cages at room temperature (26°C) in light conditions, with food (Phokphan Hi-Provite) and water to drink available *ad libithum.* The rats were obtained from Biokimia Laboratories, Medical Faculty, Airlangga University. The number of rats obtained was calculated using the formula by Frederer: Sample = (r-1) (t-1), so that the total number of rats was 48. In each cage there were 7 rats and the rats were acclimatised to their cages for 1 week. The rats were kept in cages containing clean husk cushions and covered with a cover made of tenuous wire to function air circulation. Chaff was replaced three times a day to keep the cage clean.

### Treatment

The 48 Wistar strain rats were divided into eight groups (6 rats/group/time point): Control group, treated with aquadest (no drilling); negative control group, drilling only with no treatment; P1, treated with 0.625 μg Ca(OH) _2_ (Dycal from Dentsply); and P2, treated with a combination of propolis extract (natural raw bee) propolis extract powder (from *Mellifera apis*; concentration of 0.937 μg) with Ca(OH) _2_ (0.781 μg) in a ratio of 1:1.5.

Animals were randomly allocated to each group with random number generator. For each group, rats were given a number between 1 and 48. The first 6 numbers generated were in the positive control group, the next 6 in the negative control group and so forth.

On day 1 of the experiment, all groups of experimental animals were prepared for grade 1 cavity with a low speed round bur (0.84 mm diameter) to reach the right mandibular M1 pulp chamber. Rats were anesthetized using ketamin (ket A) dissolved in a sterile isotonic saline solution (0.2 ml/87 mg bb). The treatments were applied to the molar teeth of the rats as follows: group P1, Ca(OH) _2_ base was mixed with catalyst until it formed a paste-like consistency and this was applied to the molar teeth of the animal when it received a plastic filling; group P2, propolis extract powder was mixed with Ca(OH) _2_ powder and liquid until it formed a paste-like consistency.

On day 7, half the animals from each group (6/group) were anesthetised with ether and decapitated. Their jaws were removed and placed in a sample pot with 10% formalin buffer (pH 7.4). On day 14, the remaining animals (6/group) underwent the same procedures.

### Immunohistochemistry

The dental tissue removed from the rats were kept in the sample pot with 10% formalin buffer (pH 7.4) for 24 hours. Subsequently, the tissue was decalcified using EDTA 10% at 37°C, which was replaced every day until the soft tissue broke down (this took up to 2 months). If the teeth were soft enough to be pricked with needle, then it is processed to make a paraffin block through the stages of dehydration, clearing, impregnation and embedding, as follows: dehydration, the dental tissue is kept in various percentages of alcohol (70, 80, 95, 96, 96, 96%) for 2 hours each percentage; clearing, dental tissue is placed in n-xylol for 4 hours; impregnation, the dental tissue is placed in solid paraffin for 4 hours; embedding, the dental tissue in the solid paraffin is placed into a mould base using tweezers. The mould base is filled with tissue on the cold plate and paraffin block ready to be cut. Using a microtome, tissue slices at a thickness of 0.5 cm were cut. These were stained with haematoxylin to look for macrophages, and were then prepared to check expressions of cytokines using the following kits: IL-10 (sc-52560 from Santa Cruz Biotechnology (Europe), VEGF (sc-7279 from Santa Cruz Biotechnology (Europe), IL-8 (BS3479 from Bioworld Technology), TGF-β (ab27969 from Abcam) and TLR-2 (pAb anti-TLR2 NB 200-536 from Novus Biologicals). Expression was looked at under a light binoculer microscope (Merk Olympus) at 400x magnification. For macrophages expressing the cytokines, cells that were brown were counted as positive.

### Data analysis

Data analysis was completed with ***SPSS 16.0 version***. Normality testing was done using Kolmogorov-Simrnov test, and a homogeneity test using Levene’s test. One-way ANOVA test used to assess the difference between groups, followed by post-hoc least significantt difference tests used for homogeneous data and Tamhane test used for non-homogeneous data. P value >0.05 was considered significant ^39, 40^.

## Results

The control group that was not treated with either Ca(OH) _2_ or propolis had higher VEGF expression than IL-10, TGF-β, IL-8, and TLR-2, while the negative control group (drilled but no treatment) had higher IL-8 expression than IL-10, TGF-β, VEGF, TLR-2. In the P1 group (Ca(OH) _2_ only) had higher VEGF expression than IL-8, TLR- 2, IL-10, TGF-β, while in group P2 (Ca(OH) _2_ and propolis combination) had higher IL-10 expression compared to VEGF, IL-8, TGF-β, and TLR-2 ( Table 1).

**Table 1.** IL-10, VEGF, TGF-β and TLR-2 expression in rats treated with various concentrations of propolis and calcium hydroxide for 7 days.

Group (mean ± SD)	IL-10	VEGF	IL-8	TGF-β	TLR-2
Control (aquadest)	8.83±0.75	9.16±2.71	6.16±0.98	6.33±0.81	3.50±1.04
Drilling only (negative control)	4.66±1.63	4.00±1.89	14.16±2.78	4.00±0.89	5.50±1.04
Ca(OH) _2_ only (P1)	8.67±1.86	10.50±0.54	9.00±1.67	7.83±1.16	8.16±0.75
Ca(OH) _2_ and propolis (P2)	15.00±2.36	14.83±1.47	9.83±0.98	10.66±1.36	11.16±1.16

Data were normally distributed, and homogeneity testing showed that IL-10 and VEGF were not homogeneous, while IL-8, TGF -β, TLR-2 were homogeneous. To see a significant difference using the One-way ANOVA and post-hoc testing showed that there were significant differences between the expression of IL-10, VEGF, IL-8, TGF-β and TLR-2 in the negative control group and the P2 group (combination of propolis extract with Ca(OH) _2_) (p = 0.00).

The control group (not treated) had IL-10 expression higher than VEGF, TGF-β, IL-8, and TLR-2, while the negative control group (drilled but not treated) had a higher expression of IL-8 than IL-10, TGF-β, VEGF, or TLR-2. In group P1 (Ca(OH) _2_ only) had higher IL-8 expression than VEGF, TLR-2, IL-10, TGF-β, while in P2 group (Ca(OH) _2_ and propolis combination) had higher VEGF expression compared to IL-10, IL-8, TGF-β, and TLR-2 ( Table 2).

**Table 2.** IL-10, VEGF, TGF-β and TLR-2 expression in rats treated with various concentrations of propolis and calcium hydroxide for 14 days.

Group	IL-10	VEGF	IL-8	TGF-β	TLR-2
Control (aquadest)	9.00±1.41	8.50±2.07	7.16±2.13	8.33±0.81	2.50±0.54
Drilling only (negative control)	4.67±2.58	4.67±2.42	16.50±2.25	5.67±1.03	4.50±1.04
Ca(OH) _2_ only (P1)	9.83±0.75	10.83±0.75	11.83±1.32	9.83±1.1	7.5±0.54
Ca(OH) _2_ and propolis (P2)	16.00±2.19	17.16±3.06	8.85±0.75	13.16±1.72	10.5±1.04

Data were normally distributed, and homogeneity testing showed that showed that VEGF and IL-8 expression were not homogeneous and IL-10, TGF-β and TLR-2 were homogenous. One-way ANOVA and post-hoc testing showed that there were significant differences between IL-10, VEGF, IL-8, TGF-β, and TLR-2 expression in the negative control group compared with the P2 group (combination of propolis extract with Ca(OH) _2_) (p = 0.00).

## Discussion

This study used a combination of propolis and Ca(OH) _2_ as ingredients for the formation of reparative dentin. Propolis has advantages as an anti-inflammatory ingredient as it contains CAPE ^13, 14^. The anti-inflammatory properties possessed by propolis can reduce inflammation in the pulp chamber and can induce reparative dentin ^15^. To find out whether propolis can be used as a bioproduct for stimulation of reparative dentin formation, this study tested the expression of the following cytokines: TLR-2, a membrane receptor that facilitates signaling pathway in the reparative dentin process ^16^; IL-10, an anti-inflammatory cytokine ^17, 18^; VEGF, a protein that helps in the process of angiogenesis ^19, 21^; IL-8, a pro-inflammatory cytokine that can induce the formation of reparative dentin through the recruitment of neutrophils to repair damaged tissue damage ^22, 23 ^; and TGF-β, a regulator of cell proliferation, differentiation and reparative dentinogenesis ^24^.

This study showed that on day 7, the group treated with a combination of propolis and Ca(OH) _2_ showed significantly increased expression of IL-10, VEGF, IL-8, TGF-β, and TLR-2 compared with the negative control. IL-10 expression was the highest among all the cytokines. Propolis has anti-inflammatory components, such as caffeic acid, which can inhibit the synthesis of eicosanoids from arachidonic acid and suppress the activity of COX-1 and COX-2 enzymes. Therefore, it inhibits the release of inflammatory mediators such as PGE-2 (prostaglandin), leukotrienes and thromboxanes, leading to the increasing expression of IL-10 expression with increasing dose of propolis ^23, 24^. The decrease in prostaglandin is caused by the inhibition of prostaglandin synthesis from arachidonic acid which is catalyzed by the transformation of the cyclooxygenase enzyme, which is produced by galangin compounds from flavonoids and CAPE. In addition, galangin from flavonoids are also able to inhibit leukotrienes from arachidonic acid through the enzyme lipoxygenase ^25, 24^.

The study showed that on day 14, there were changes in the expression of IL-10, TGF-β, and TLR-2 in the combination treatment group compared with the negative control group. The expression of IL-10, TGF-β, and TLR-2 in the treatment group treated with Ca(OH) _2_ alone without propolis was more significant compared with the negative control, whereas the expression of VEGF and IL-8 in the treatment group treated with a combination of propolis and Ca(OH) _2_ was significantly increased compared with the negative control. The combination treatment group showed the highest VEGF expression among other variables, while the Ca(OH) _2_ group showed that the expression of IL-10 was highest among the other variables. The 14th day is probably when the process of proliferation and angiogenesis has been initiated. In this research combination therapy of propolis and calcium hydroxide increases VEGF at 14 days. This could be due to the role of PDGF (platelet-derived growth factor), which is known as a promoter of proteoglycan and collagen formation. As local fibroblasts respond to PDGF by producing collagen and transforming it into myofibroblasts to increase wound contraction, fibroblasts also emit keratinocyte growth factor (KGF), which stimulate epithelialization from keratinocytes, and produce VEGF endothelial cell growth factors, and fibroblast growth factors (bFGF) to promote blood vessel growth ^27^. IL-10 expression has also increased because Ca(OH) _2_ has high alkaline properties, which means that it will increase the pH for pulp healing if damaged as a high pH can neutralize acids in the inflammatory site ^28^.

Ca(OH) _2_ is used as an intracanal medicament and is antibacterial. Ca(OH) _2_ is used because it is stable for a long time and can kill bacteria ^26, 29^. In previous studies, endodontic therapy using Ca(OH) _2_ showed that IL-1α as an indicator of pro-inflammatory cytokines decreased on day 7, indicating that there is an immune system response to reduce the inflammatory process so that the expression of IL-10 as an anti-cytokine -inflammation will increase ^26, 30^.

Calcium hydroxide can induce odontoblast formation through increased VEGF, this is due to the occurrence of differentiation and viability of endothelial cells ^31, 32^. VEGF is also expressed by odontoblasts and sub-odontoblasts by large numbers of endothelial cells ^33^. VEGF also has a key role in the physiological and pathological development of dentinogenesis and angiogenesis in healthy dental pulp ^33, 34^. In previous studies, odontoblast-like cells and undifferentiated pulp cells express high amounts of VEGF in vitro ^35^. In the inflammatory process, day 7 shows an increase in VEGF after the formation of granulation tissue and decreases until day 14 ^35, 36^. The VEGF mechanism is as an angiogenesis process through odontoblasts; LPS stimulated from bacteria present in the pulp can increase the permeability of the blood vessels so as to facilitate the process of neutrophil, lymphocyte and monocyte diapedesis and recruit new blood vessels around the carious tissue to increase immune defense ^37^.

The reparative dentin stimulation process occurs after pulp damage which is then treated using pulp capping material in the form of calcium hydroxide Ca(OH) _2_ which is influenced by the nature of cytotoxicity and cytokine production ability ^34, 35^. Inflammatory cytokines such as IL-8 found in dental pulp will recruit neutrophils in case of infection from bacteria, which will cause acute inflammation. IL-8 also plays a role in determining the duration of the inflammatory process ^21^.

In the combination treatment of propolis with Ca(OH) _2_ from the 7th day to the 14th day, the expression of IL-10, VEGF, TGF-β increased while IL-8 and TLR-2 decreased. Propolis activated the initial steps of the immune response by upregulating TLR-2 expression and the production of pro-inflammatory cytokines in the rats, modulating the mechanisms of innate immunity. TGF-β1 may be involved in the healing/regeneration processes of dental pulp in response to injury by stimulation of collagen and TIMP-1 production ^37^. These events are associated with activin receptor-like kinase-5, Smad2/3 and MEK/ERK signaling and TGF-β will inhibit TLR-2 mediated odontoblast pathway when inflammation occurs in the pulp chamber ^37, 38^. TGF-β is a signaling molecule that induces cell proliferation, chemotaxis and apoptosis in monocyte, epithelial, mesenchymal and neuron tissues. In the formation of reparative dentin, TGF-β has an important role in regulating cell proliferation, differentiation and reparative dentinogenesis ^23^. The release of TGF-β from the dentinal matrix requires diffusion, which generally passes through the dentinal tubules to the pulp cell to activate the signaling pathway through TLR-2. Activation of TLR-2 will increase the effector of the innate immune system, including pro-inflammatory cytokines, chemokines, and anti-inflammatory cytokines to repair tissue damage ^39^. This is consistent with the results of this research showing that with the combination of propolis with Ca(OH) _2_ TGF-β expression is higher than in the treatment group with only Ca(OH) _2_. TGF-β is secreted by odontoblasts, and its expression is increased in carious lesions. TGF-β is proinflammatory during the initial stages of inflammation, and anti-inflammatory in later stages. Proinflammatory effects of TGF- β include immune cell recruitment and induction of matrix metalloproteinase secretion, and stimulation of the accumulation of immature dendritic cells in odontoblast and subodontoblast layers of the pulp horn close to the lesion in strategic locations to encounter foreign antigens entering the dentinal tissue ^14, 39^.

IL-8 is expressed continuously by odontoblast cells. IL-8 plays a role in neutrophil recruitment in cases of inflammation in the pulp to support the healing process ^39^. In this study, IL-8 was expressed at a lower in the propolis and Ca(OH) _2_ combination treatment group compared with the group treated with Ca(OH) _2_ only. This illustrates that the administration of a combination of propolis and Ca(OH) _2_ can repair pulp tissue damage through the formation of reparative dentine. On the 14th day with propolis and Ca(OH) _2_ combination, VEGF expression was high so that it can help the process of angiogenesis in the dental pulp to be faster, while the group with only Ca(OH) _2_ has anti-inflammatory properties and high IL-10 expression so as to reduce inflammation which occurs in the pulp chamber.

## Conclusion

Combination therapy of propolis with Ca(OH) _2_ could be considered as a product for pulp capping treatment that can stimulate the formation of reparative dentin.

## Data availability

### Underlying data

Harvard Dataverse: Combinations of propolis and Ca(OH) _2_ in dental pulp capping treatment for the stimulation of reparative dentin formation in a rat model, https://doi.org/10.7910/DVN/SLV60A ^41^

This project contains the following underlying data:

- Values for immunohistochemistry for all groups for 7-day treatment
- Values for immunohistochemistry for all groups for 14-day treatment
- **Figure S1. Mean of IL-10, VEGF, IL-8, TGF-β and TLR-2 expressions on the 7th day**.
- **Figure S2. Mean of IL-10, VEGF, IL-8, TGF-β and TLR-2 expressions on the 14th day**.
- **Figure S3. Immunohistochemical examination results from VEGF.** A. Control, B. Negative Control, C. Therapy with Ca(OH) _2_, D. Therapy with a combination of propolis and Ca(OH) _2_.
- **Figure S4. Results of immunohistochemical examination of IL-10.** A. Control, B. Negative Control, C. Therapy with Ca(OH) _2_, D. Therapy with a combination of propolis and Ca(OH) _2_.
- **Figure S5. Immunohistochemical examination results from TGF-β.** A. Control, B. Negative Control, C. Therapy with Ca(OH) _2_, D. Therapy with a combination of propolis and Ca(OH) _2_.
- **Figure S6. Results of immunohistochemical examination of TLR-2.** A. Control, B. Negative Control, C. Therapy with Ca(OH) _2_, D. Therapy with a combination of propolis and Ca(OH) _2_.
- **Figure S7. Results of immunohistochemical examination of IL-8.** A. Control, B. Negative Control, C. Therapy with Ca(OH) _2_, D. Therapy with a combination of propolis and Ca(OH) _2_.

Data are available under the terms of the Creative Commons Zero "No rights reserved" data waiver (CC0 1.0 Public domain dedication).
