# Supplementary material for: Combinations of propolis and Ca(OH)2 in dental pulp capping treatment for the stimulation of reparative dentin formation in a rat model
Source: F1000Res. 2020 Apr 29;9:308. [Version 1] doi: 10.12688/f1000research.22409.1 (PMC7369428; doi:10.12688/f1000research.22409.1)
Supplement: Peer review history

# Peer review: Reviewer response for version 1

**DOI:** 10.5256/f1000research.24725.r63240

**Published:** 2020-07-17

**Authors:** Noengki Prameswari

**Affiliations:** 1 Orthodontic laboratory Staff, Dentistry Faculty, Hang Tuah University, Surabaya, Indonesia

1. The sample size mentioned in abstract methods and full-text methods are very vague. They should be rewritten for better clarity. 
2. Methods: you mention that this treatment for stimulating the formation of reparative dentine. What is the clinical parameter for showing that your treatment (dental pulpcapping) has an effect on the formation of reparative dentine?
3. Discussion: The explanation about the role of natural ingredient in Propolis and its combination with Ca(OH) _2_ as dental pulpcapping to stimulate the formation of reparative dentine is not clear.
4. Conclusion: you should state the difference result of pulpcapping treatment in 7 days and 14 days, and also the mechanism.  

Is the work clearly and accurately presented and does it cite the current literature?

Yes

If applicable, is the statistical analysis and its interpretation appropriate?

Yes

Are all the source data underlying the results available to ensure full reproducibility?

Yes

Is the study design appropriate and is the work technically sound?

Yes

Are the conclusions drawn adequately supported by the results?

Partly

Are sufficient details of methods and analysis provided to allow replication by others?

Yes

Reviewer Expertise:

Bone remodeling, herbal medicine, physiology, orthodontic

I confirm that I have read this submission and believe that I have an appropriate level of expertise to confirm that it is of an acceptable scientific standard.

# Peer review: Reviewer response for version 1

**DOI:** 10.5256/f1000research.24725.r65377

**Published:** 2020-07-06

**Authors:** Mohd Farhan Hanif Reduan

**Affiliations:** 1 Department of Paraclinical Studies, Faculty of Veterinary Medicine, Universiti Malaysia Kelantan, Kota Bharu, Malaysia

**Reviewer comment:**

 Combinations of propolis and Ca(OH)2 in dental pulp capping treatment for the stimulation of reparative dentin formation in a rat model.

**Reviewer comments:**

 In general, the research is well conducted and the findings are worth considering for publication. The paper needs a minor revision as below:

- Conclusion of the abstract, please omit the “antibacterial property” as the research conducted not based on the antibacterial study.
- Paragraph 1: *“NF-kB activation due to injury causes transcription and translational processes in cells, which results in pro-inflammatory cytokine production, such as IL-1β, IL-6 and TNF-α, initiating the immunological process that aids pulp tissue in overcoming inflammation*” - the reviewer suggest confirming this statement as pro-inflammatory cytokines such as IL-1β, IL-6 and TNF-α are promoting inflammation, not overcome the inflammation, so need clarification on this statement.
- Methodology – in the animal section, the authors need to replace the “biokimia” term into the English term.
- Methodology – in the treatment section, the authors need to highlight the experiment is separated into two periods of time; 7 and 14 days at the initial, easier for the reader to capture, the authors need to explain why two different concentrations of calcium hydroxide were selected in the discussion.
- Methodology – in the immunohistochemistry section, please use a past tense to describe the terms, few in the present tense, the authors need to explain in detail on the evaluation of the expression of cytokines like the intensity of the brownish expression and etc. If possible, include references on the method of immunohistochemistry. It is because immunohistochemistry is a major part of this study.
- Results – The authors are suggested to include the statistical indication in Tables 1 and 2. It will help the reader to understand the table faster rather than read in the sentences. Please put arrows in the figures.
- Discussion – Paragraph 2; *“In addition, galangin from flavonoids are also able to inhibit leukotrienes from arachidonic acid through the enzyme lipoxygenase”- *authors need to state what is the source of galangin, is it from propolis?
- Discussion – Paragraph 3; “The 14th day is probably when the process of proliferation and angiogenesis has been initiated”, need to improvise the statement as the proliferation and angiogenesis are at the peak in the 14 ^th^ day.
- Discussion – Paragraph 5: *“Ca(OH) _2_ is used as an intracanal medicament and is antibacterial. Ca(OH) _2_ is used because it is stable for a long time and can kill bacteria”. *The authors are suggested to further discuss the details on the effect of calcium hydroxide as caries always related to bacterial infection.
- Discussion – Paragraph 8: The authors need to add elaboration on the anti-inflammatory activity of TGF at the late stage.
- Conclusion – the mechanism on the stimulation of the formation of reparative dentin needs to be addressed based on the present findings.

Is the work clearly and accurately presented and does it cite the current literature?

Yes

If applicable, is the statistical analysis and its interpretation appropriate?

Yes

Are all the source data underlying the results available to ensure full reproducibility?

Yes

Is the study design appropriate and is the work technically sound?

Yes

Are the conclusions drawn adequately supported by the results?

Yes

Are sufficient details of methods and analysis provided to allow replication by others?

Yes

Reviewer Expertise:

My research on the wound healing and toxicological study using an animal model in particular to pathological evaluation.

I confirm that I have read this submission and believe that I have an appropriate level of expertise to confirm that it is of an acceptable scientific standard, however I have significant reservations, as outlined above.

# Peer review: Reviewer response for version 1

**DOI:** 10.5256/f1000research.24725.r62925

**Published:** 2020-05-07

**Authors:** Guang Hong

**Affiliations:** 1 Liaison Center for Innovative Dentistry, Graduate School of Dentistry, Tohoku University, Sendai, Japan

This paper needs minor revision. I would make the following comments.

**Introduction** **:**

1. Please add the hypothesis of your study in this section.

**Methods:**

1. In your study, you use the 16-18 weeks rat. I think it is a little bit old, how do you think? Please add the reason why you choose this age rat in the discussion section.

**Discussion** **:** 

1. The answer to the hypothesis of this study should be included in the discussion section.
2. Natural ingredients are difficult to obtain stable properties. Please add the discussion regarding this issue in the discussion section.
3. Please add the clinical implications of your study and the clinical significance of the findings in the discussion section.
4. What is the limitation of this study? You should indicate in this section.

**Figure** **:**

1. Fig. 1 and Fig. 2: Please add the result of the statistical analysis.
2. Fig. 3 to Fig. 7: Please use arrows to indicate the point that you want to explain.

Is the work clearly and accurately presented and does it cite the current literature?

Yes

If applicable, is the statistical analysis and its interpretation appropriate?

Yes

Are all the source data underlying the results available to ensure full reproducibility?

Yes

Is the study design appropriate and is the work technically sound?

Yes

Are the conclusions drawn adequately supported by the results?

Yes

Are sufficient details of methods and analysis provided to allow replication by others?

Yes

Reviewer Expertise:

Biomaterials and tissue engineering

I confirm that I have read this submission and believe that I have an appropriate level of expertise to confirm that it is of an acceptable scientific standard, however I have significant reservations, as outlined above.
